# Two-Dimensional LiDAR Sensor-Based Three-Dimensional Point Cloud Modeling Method for Identification of Anomalies inside Tube Structures for Future Hypersonic Transportation

**DOI:** 10.3390/s20247235

**Published:** 2020-12-17

**Authors:** Jongdae Baek

**Affiliations:** Future Infrastructure Research Center, Korea Institute of Civil Engineering and Building Technology (KICT), Goyang 10223, Korea; jdbaek@kict.re.kr; Tel.: +82-31-910-0754

**Keywords:** LiDAR, point cloud, anomaly detection, tube structure, future transportation, safety monitoring

## Abstract

The hyperloop transportation system has emerged as an innovative next-generation transportation system. In this system, a capsule-type vehicle inside a sealed near-vacuum tube moves at 1000 km/h or more. Not only must this transport tube span over long distances, but it must be clear of potential hazards to vehicles traveling at high speeds inside the tube. Therefore, an automated infrastructure anomaly detection system is essential. This study sought to confirm the applicability of advanced sensing technology such as Light Detection and Ranging (LiDAR) in the automatic anomaly detection of next-generation transportation infrastructure such as hyperloops. To this end, a prototype two-dimensional LiDAR sensor was constructed and used to generate three-dimensional (3D) point cloud models of a tube facility. A technique for detecting abnormal conditions or obstacles in the facility was used, which involved comparing the models and determining the changes. The design and development process of the 3D safety monitoring system using 3D point cloud models and the analytical results of experimental data using this system are presented. The tests on the developed system demonstrated that anomalies such as a 25 mm change in position were accurately detected. Thus, we confirm the applicability of the developed system in next-generation transportation infrastructure.

## 1. Introduction

To achieve hyperconnectivity in line with the fourth industrial revolution (4IR) and solve various social issues caused by urbanization worldwide—such as population concentration, traffic congestion and accidents, and environmental problems—the hyperloop transportation system has emerged as an innovative next-generation transportation system. In this system, the inside of a sealed transport tube is brought to a near-vacuum state to reduce air resistance; in this tube, a capsule-type vehicle moves at 1000 km/h or more using a magnetic levitation propulsion system. Not only should this transport tube span long distances, it must also not tolerate even minor potential dangers to vehicles traveling at high speeds inside it. Therefore, an automated real-time infrastructure anomaly detection system for monitoring the safety of the tube structure is essential.

While visual inspection and nondestructive technologies are typical diagnostic tools for the structure’s exterior, visual diagnosis of a target structure’s local area or surface is unsuitable if the structure is large. In particular, visual and nondestructive inspections performed manually by humans are labor-intensive and may reflect the subjective opinions of the inspectors based on their experience. Moreover, inspections can be limited depending on the location and conditions of the facility, and it is difficult to respond to sudden crises because inspections are commonly conducted on a fixed schedule. Recently, researchers performed numerous studies on noncontact detection techniques to survey and analyze discontinuities in SOC (Social Overhead Capital) facilities. Consequently, technologies and products that determine a structure’s conditions using this information are emerging. Earlier common manual survey techniques are inadequate for representing the overall conditions of a facility because they only sample a specific area. Hence, it is difficult to predict structural defects using such techniques.

Complete enumeration surveys that inspect an entire long-distance facility, such as a hyperloop tube, are laborious. In addition, integrated data are unreliable because of the low consistency of measurement results which are conducted by many surveyors. Therefore, precise and rapid detection technologies are necessary for facilities with poor accessibility, especially those in a near-vacuum state such as hyperloop tubes or those that require the expeditious acquisition of data.

Researchers are developing imaging diagnostic technology that can address the limitations of existing nondestructive inspection techniques. To effectively analyze and interpret object information, three-dimensional (3D) shape-measurement technology that extracts and realizes the geometric spatial information of an object is required. Researchers have developed and implemented a variety of contact and noncontact measurement techniques in practice. Although contact measurement techniques have excellent precision, they suffer from certain problems: the measurement time is long and the sensitivity to the area outside the measurement point decreases. Noncontact measurement techniques are utilized in many fields because they enable high-speed measurement and a high density of measurement points owing to recent technological advances.

Light Detection and Ranging (LiDAR) is an active noncontact distance measuring (ranging) system that functions by illuminating the target with a laser beam and sensing the reflected laser light. Because of its high accuracy, LiDAR is used in various fields such as surveying, geology, atmosphere, industry, etc. [1,2,3,4,5,6,7,8,9,10]. There has recently been a surge in research on facility-related detection systems using LiDAR. For example, Verma et al. [1] presented a method of detecting and constructing a 3D model of an urban area using aerial LiDAR. Chen et al. [2] tried to reconstruct power pylons using airborne LiDAR data. Yang et al. [3] suggested a method for detecting road markings from noisy point clouds generated by LiDAR scanning.

Research interest in noncontact rapid detection technologies that actively use laser scanners and imaging cameras has also increased. Kuroki et al. [11] conducted experiments on object identification with shape discrimination by configuring a range image sensor using a multislit laser projector and a CCD camera. The empirical results showed that using a line laser, rather than a multispot projector as previously studied, greatly improved the scanning performance. Kuroda et al. [12] developed a laser scanning-type 3D dental cast analyzing system using a slit-ray laser beam and a CCD camera. They created a driving structure that could freely move the laser beam and the CCD camera to enable scanning in narrow spaces and describe the scanning of objects from various angles. Tsai [13] defined the relationship between the real-world coordinate system and the camera coordinate system using the coplanar calibration method. They calculated the mapping transformation between the real-world coordinate system and camera coordinate system as a determinant, and proposed a method for easily transforming the matrix according to the camera change. Levoy et al. [14] and Rocchini et al. [15] conducted experiments by generating 3D profiles using measuring instruments that scan solid objects in three dimensions.

Just as LiDAR and image recognition techniques are being applied to 4IR technologies, such as autonomous vehicles [16,17,18] and drones, noncontact object detection technology is currently being applied to infrastructure [19,20,21,22]. The use of LiDAR technology for infrastructure maintenance inspection sometimes requires point cloud comparison methods. De Asís López et al. proposed a method for point cloud comparison [23].

To automate anomaly detection in long-distance facilities such as hyperloops, this study used a method of generating 3D point cloud models of facilities with a vehicle-mounted two-dimensional (2D) LiDAR-based measurement system. This study attempted to detect facility anomalies using an algorithm that compares and identifies changes between a reference 3D model of the facility in its initial state without any change and an experimental model generated by periodic measurements for monitoring purposes. First, the sensor system construction method and the system creation process are presented, followed by the data generation method and data verification procedure. Finally, the experimental results are analyzed, and the applicability of the proposed anomaly detection system is described.

Hyperloops are different from maglevs (although they use passive maglevs) and all other existing transportation modes, in that hyperloops use closed tubes in near-vacuum states in which pod-type vehicles move at 1000 km/h or more. This study provides a method that can be used to implement an automated 3D safety monitoring system, which is a significant step in the development and realization of hyperloop transportation.

## 2. Proposed Framework for Automatic Infrastructure Anomaly Detection

**Figure 1:**
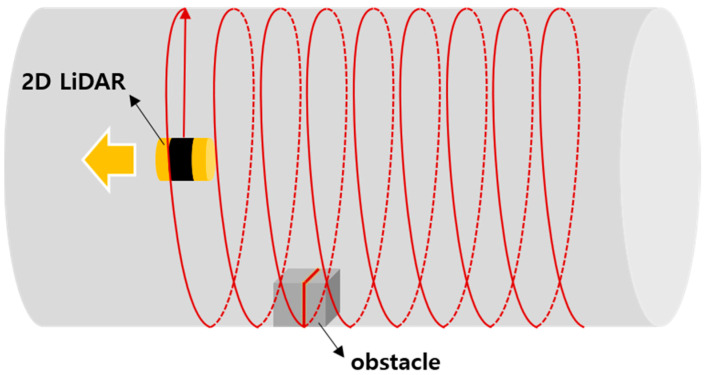
Basic conceptual diagram of two-dimensional (2D) Light Detection and Ranging (LiDAR) scanner moving in a tube facility for inspection.

This study attempted to develop a system that enables safety monitoring by automatically and rapidly detecting anomalies in long-distance tunnel-tube structures such as hyperloop tubes, including out-of-place internal installations and foreign objects. In a hyperloop, as the capsule moves at a high velocity of 1000 km/h in a near-vacuum tube, it is crucial to detect any potential hazards that may affect the vehicle in advance. In this study, as shown in Figure 1, a method by which 3D point cloud data are obtained from a 2D LiDAR scanner that rotates and moves in the tube facility and generates 3D models of the internal shape of the tube to determine the presence of anomalies was adopted.

**Figure 2:**
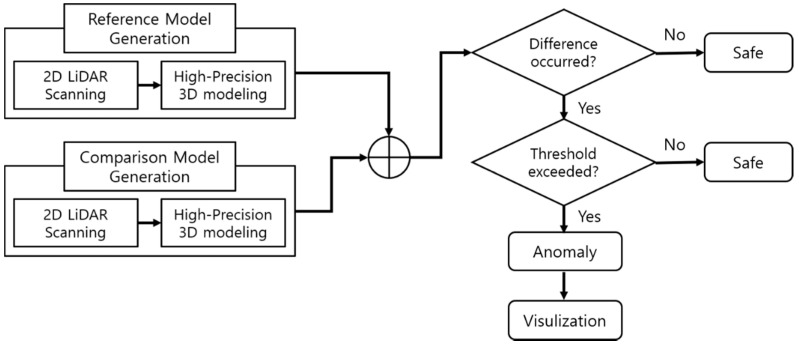
Flow chart of facility anomaly detection.

Figure 2 is a flow chart of facility anomaly detection using this concept. First, a moving 2D LiDAR sensor was used to scan the target facility in order to acquire 3D data to generate a 3D point cloud model of the facility in a normal state; this model was used as a reference model for comparison. Next, a comparison model was generated using the same method that was used for the reference model. The comparison model was intended to simulate a situation in which an anomaly occurred inside the facility, under the assumption that this type of scenario would probably occur in real conditions. Then, the presence of anomalies in the facility was detected through an algorithm that compares the two models and indicates abnormalities when the difference is above a certain threshold, which is set by the user. Finally, the results were shown with a visualization tool.

A 3D point cloud is a coordinate system set (*x*, *y*, *z*) of data points; it typically displays the attribute values as *x*, *y*, *z* values of the Cartesian coordinate system to express the outer contours or surfaces of the target object. An object’s shape is expressed by collecting its 3D values, and the difference in each attribute value can be used to determine whether the object has changed. Point clouds can be generated by laser scanners such as LiDAR; these devices can automatically measure multiple points on an object’s surface and model the object in the format of a data file.

**Figure 3:**
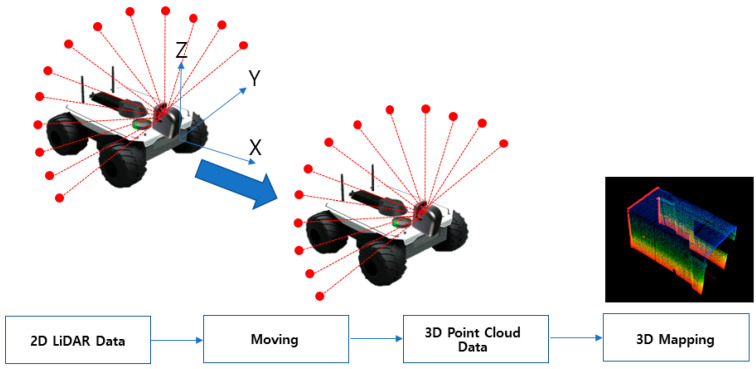
Three-dimensional mapping using 3D point cloud.

Figure 3 illustrates the concept of 3D mapping using 3D point cloud data. By mounting the 2D LiDAR sensor on a mover and then shuttling it, the 3D point cloud data (*x*, *y*, *z*) can be acquired using the position data obtained from the encoder and the IMU (Inertia Measurement Unit) of the mover. The scan matching technique was then used to perform 3D mapping with the 3D point cloud dataset. A reference model, and the comparison models generated thereafter via high-precision 3D mapping, were compared to determine whether the comparison models exceeded the threshold value using the normal distribution transform (NDT) matching technique.

The constructed 3D reference model data of a transport tube and the subsequent periodically measured 3D model data were compared. The feature data representing the changes in the tube’s condition were generated using this comparison. The facility anomaly detection system had functionalities such as provision of tube infrastructure information including gradients, color coded values for magnitudes above the threshold, and a warning alarm in the event of change detection.

## 3. Two-Dimensional LiDAR Sensor-Based Three-Dimensional Safety Monitoring System Prototype

### 3.1. Two-Dimensional LiDAR Sensor Prototype

This study introduces a method of generating a 3D model of the space inside the tube using point cloud data to quickly and accurately monitor and detect anomalies in this space. The point cloud data are collected using a 2D LiDAR scanner. As the rotation frequency and angular resolution of LiDAR are important when using point clouds to generate 3D models of long-distance linear structures such as tubes, and considering the possibility of improving the performance of those LiDAR sensors, this study chose a 2D LiDAR sensor system instead of the existing 3D LiDAR sensors. The existing 3D LiDAR sensors have several disadvantages, especially for this purpose, because of structural aspects including rotation frequency and signal processing bottleneck. For this study, a customized 2D LiDAR sensor prototype with enhanced rotation frequency and angular resolution performance was built.

The sensor system built for this experimental study was a medium-range 2D LiDAR that used the time-of-flight method. The method provides 2D spatial information for a specific range using a scanning point beam. The 2D LiDAR sensor is applicable to robots, drones, and industrial automation and is used in practice across industrial fields.

The laser transmitter uses IR rays with a wavelength of 905 nm, which can be viewed via an IR card or IR camera. The trigger signal period that determines the interval of the transmission beam was 13.33 μs. Thus, the pulse repetition rate of the transmitter was 75 kHz, meaning it could emit 75,000 laser beams per second. The avalanche photodiode (APD) of the receiver was operated at a high voltage with an operating voltage of 120–220 V. A brushless DC (BLDC) motor was used to rotate the sensor, which can rotate at a frequency of 20 or 40 Hz. The angular resolution can be calculated using the trigger signal period and motor rotation frequency. As shown in Equation (1), at a rotation frequency of 20 Hz, the angular resolution is approximately 0.1°.

(1) $$angular\;resolution=motor\left(20\;Hz\right)\times 360{}^{\circ}\times \;\frac{1}{trigger\left(75\;kHz\right)}=0.096{}^{\circ}$$

**Figure 4:**
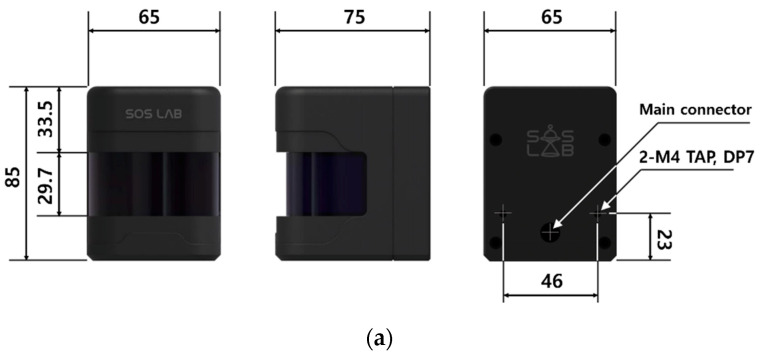

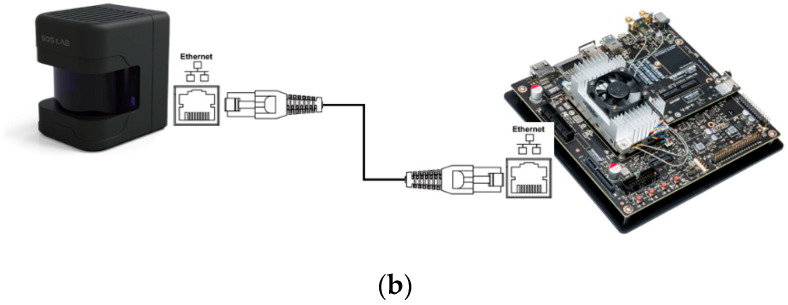
Two-dimensional LiDAR sensor exterior and LiDAR connection interface: (**a**) exterior of 2D LiDAR sensor; (**b**) 2D LiDAR connection interface.

In terms of the operating range, the device has a horizontal field of view of ±90° (total 180°) based on the rotating shaft of the motor in the main body and the front of the LiDAR, with a sensing distance of 0.05–10 m from the measurement center. The distance measurement error within the sensing distance was ±30 mm and had a repeatability of ±30 mm. A dedicated sensor operation control circuit and software were developed, and the user datagram protocol (UDP) was applied to the interface. Figure 4 shows the 2D LiDAR sensor exterior and LiDAR connection interface.

### 3.2. Robotic Transportation System Shuttling the LiDAR Sensor

**Figure 5:**
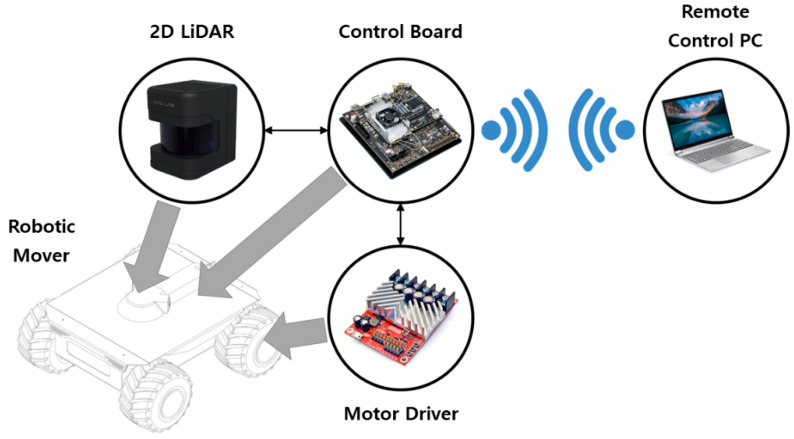
Hardware configuration of robotic transportation system.

To generate the 3D point cloud data, a robotic transportation system on which the 2D LiDAR prototype could be mounted and shuttled was manufactured. Figure 5 illustrates the hardware configuration of the robotic transportation system. The robotic transportation system was controlled by an embedded control board, and a motor driver was used to collect the mover’s encoder data and control the speed of the mover. The odometry was calculated from the acquired encoder data, which was combined with data obtained from the 2D LiDAR to generate the 3D point cloud. Control board commands and data acquisition were carried out via wireless communication from a laptop computer.

The robotic mover has a size of 426 mm (W) × 455 mm (L) × 207 mm (H) and a weight of approximately 2 kg. It is powered by a battery and has an operating time of approximately 30 min. The motor driver is embedded with a proportional-integral-derivative (PID) speed controller to enable precise motor control without the need for a subcontroller. It can achieve a maximum speed of above 2 m/s, which is also the target maximum speed. NVIDIA Jetson TX2 was used as the control board. Owing to its high-performance and low-power computing functions suitable for deep learning and computer vision, the Jetson TX2 is widely adopted in embedded projects that use extensive computation, such as drones, autonomous robot systems, and mobile medical imaging.

**Figure 6:**
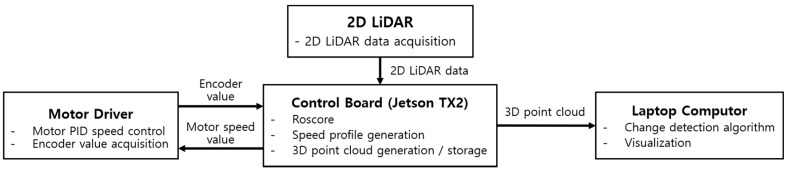
Software configuration diagram of robotic transportation system.

Figure 6 outlines the software configuration of the robotic transportation system. A robot operating system (ROS) was used to control the robotic mover, in which the following packages were installed:

- 2D LiDAR operation package: package for publishing 2D LiDAR data;
- Motor driver operation package: package for subscribing to receive the speed input of the robotic mover and publishing the encoder value;
- Robotic mover velocity profile generation package: package for generating the velocity profile of the mover and publishing data;
- 3D point cloud generation package: package for subscribing the encoder data and LiDAR data, publishing the 3D point cloud, and saving it as a PCD (Point Cloud Data) file.

**Figure 7:**
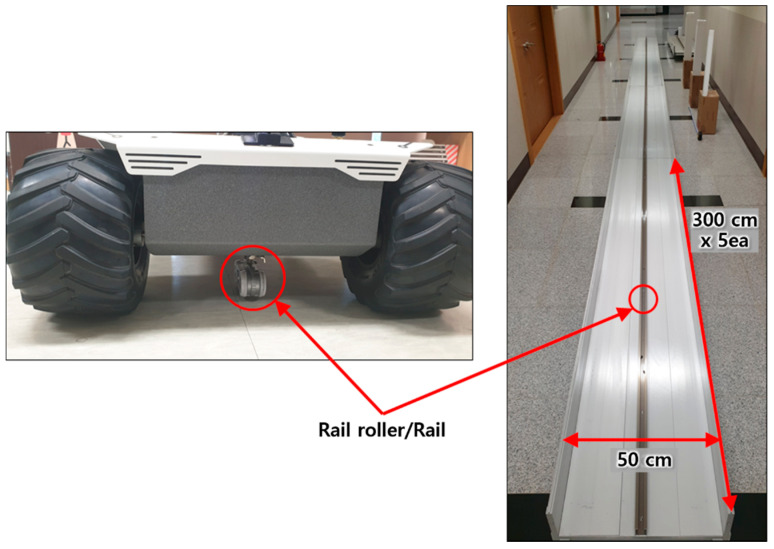
Robotic mover travel rail.

To conduct the experiments for assessing the performance of the 3D safety monitoring system, rails with a total width of 50 cm and a total length of 15 m (five rails with a length of 300 cm each) were manufactured. A rail roller was attached to the robotic mover to enable the mover to travel in a straight line along the rail without shaking. Figure 7 displays photographs of the travel rail and the rail roller for the robotic mover.

### 3.3. Three-Dimensional Safety Monitoring System Prototype

This study developed a 3D safety monitoring system prototype that determines and visualizes anomalies via an algorithm that compares 3D models generated using 3D point cloud data. The system consists of four modules: a precise 3D model generation module, a 3D model registration module, a change detection and anomaly determination module, and a sensor measurement data collection and point cloud data visualization viewer module.

First, an algorithm for generating the 3D model was designed for the 3D model generation module. A right-handed coordinate system was used for the 3D coordinate system, in which the x-axis is the movement direction and the *y*- and *z*-axes are the 2D LiDAR scanning directions. A 3D point cloud dataset was created using the movement distance of the robotic mover (*x*-axis values), calculated through the encoder data and a kinematic model as well as the 2D LiDAR data (*y*- and *z*-axes values) acquired from the 2D LiDAR prototype. This dataset was then used to create a 3D map. The 3D point cloud data were saved in the PCD file format.

**Figure 8:**
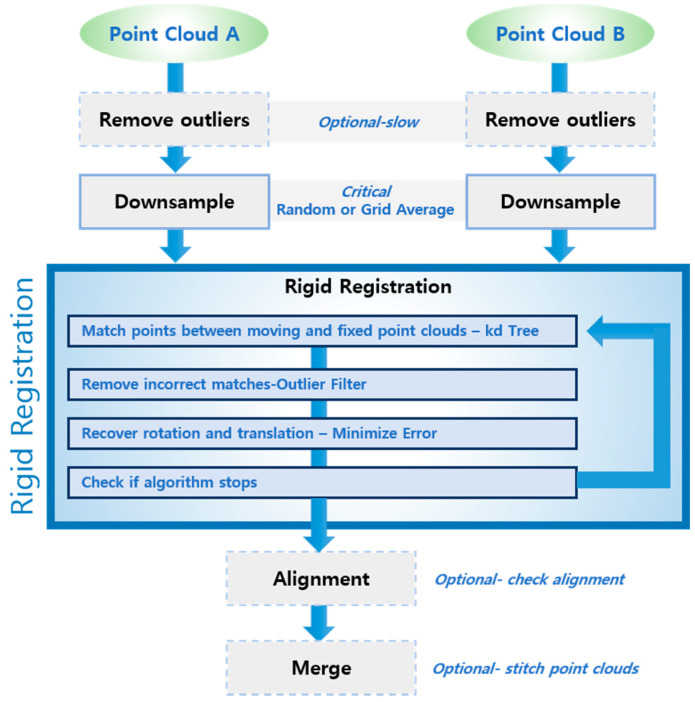
Point cloud registration workflow.

The method for detecting anomalies or environmental changes adopted in this study is as follows. First, the facility is measured beforehand in a state of no anomalies, based on which a 3D reference model is created. Its shape is then compared with that of a 3D model generated through constant monitoring during actual operation, through which any changes in the facility environment or anomalies can be detected. To compare the change in shape of the two 3D models, their orientations must first be aligned in a preliminary process. Accordingly, a point cloud registration algorithm was designed for the 3D model registration module (Figure 8). As a preprocessing step to reduce the computational cost and enhance the registration precision of the registration algorithm, the voxel-grid downsampling technique was applied to two map models to acquire point cloud data of uniform density. The targeted precision of cloud registration had a root mean square (RMS) error of 50 mm.

The registration algorithm comprises two steps (coarse-to-fine). First, feature points based on fast point feature histograms (FPFHs) are extracted from each map model, and a transformation matrix representing the geometric relationship between the two models is extracted using a random sample consensus (RANSAC)-based perspective-n-point (PnP) algorithm. FPFH is a simplified version of point feature histograms (PFHs) that reduces the computational complexity, while retaining the discriminative power of the PFH [24]. To make calculation faster, for each point, a method similar to PFH is used to calculate the triples (relationships between the point and its neighbors) and a simplified point feature histogram (SPFH) is obtained. Then, for each point, a weighted neighboring SPFH is used to calculate the final value of the histogram as follows:

(2) $$FPFH\left(p\right)=SPFH\left(p\right)+\frac{1}{k}\sum_{i=1}^{k}\frac{1}{\omega_{k}}\cdot SPFH\left(p_{k}\right),$$

where the weight ω*_k_* represents the distance between point *p* and a neighboring point *p_k_* using a given distance metric space. Once the orientation between the two map models is estimated through the transformation matrix estimated in the first step, the accuracy of the matrix is improved using an iterative closest point (ICP) registration algorithm.

Precise registration is necessary to avoid the phenomenon in which the cloud density and shape vary with the scanning frequency and speed of the mover. The cloud density also varies with the type of surface that is being scanned. The balance of the distribution of points, which is a key factor for laser scanning systems, depends on the surface uniformity. The surface scanned in this study, which is also the surface type intended for use in future transport tubes, is quite uniform, so a uniform spatial pattern is expected.

It is also needed to effectively reduce the computational cost. A preprocessing technique based on the voxel-grid downsampling was applied before aligning the orientations of the two map models. For example, approximately 1,257,000 points of point cloud data would be obtained if the original data were scanned at a rotation frequency of 20 Hz and a forward velocity of 0.3 m/s. Performing orientation registration on these data without a sampling process took an average of 58.43 s on an Intel i5-8600 with 8 GB of RAM. In contrast, by sampling the same data at 5 cm intervals, the point cloud data were reduced to 38,000–40,000 points and the orientation alignment speed improved to 0.91 s. This not only enhances the processing speed, but also the robustness of the registration algorithm, as scanned data of different densities are processed to a certain density to perform the registration. Because changes and anomalies were detected in the original data based on the aligned orientation, the resolution and inspection precision of data were retained.

**Figure 9:**
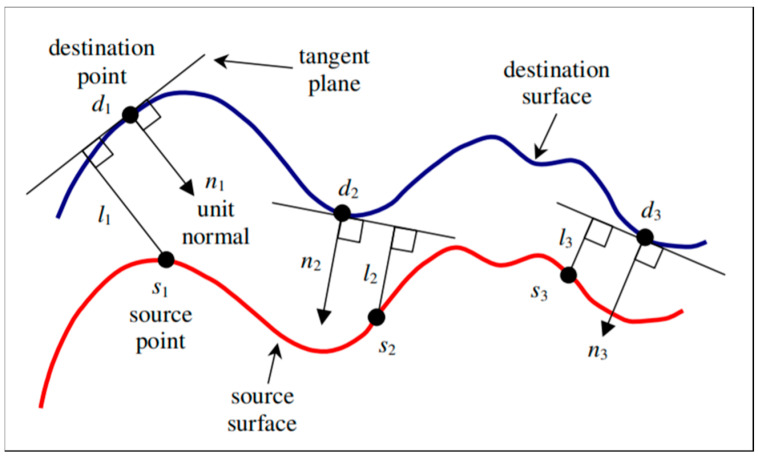
Point-to-plane projection distance.

The change detection and anomaly determination module was designed to use the point-to-plane projection distance (Figure 9), an indicator for comparing the shape changes of the aligned reference model and the comparison model. The projection distance from each point of the comparison model to the nearest plane on the reference model is calculated using Equation (3); if this value exceeds the change detection threshold, then it is judged as an abnormal change. The targeted change detection precision was within 50 mm for the *y*- and *z*-axes and within 30 mm for the *x*-axis.

(3) $$e={\left(\left(s_{i}-d_{i}\right)\cdot n_{i}\right)}^{2},$$

where *e*, *s*, *d*, and *n* represent the projection distance, source point, destination point, and unit normal vector, respectively.

Finally, the sensor measurement data collection and point cloud data visualization viewer module was used to transmit, store and load the sensor measurement data, as well as visualize the point cloud data in 3D and visualize and display the detected abnormal changes. The operating environment was Ubuntu OS, which is based on Linux.

## 4. Results and Discussion

**Figure 10:**
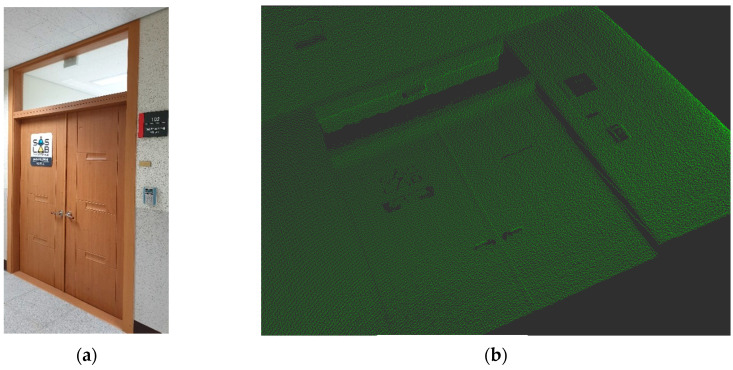
Image of the sensor test scanning result: (**a**) photograph of the scanning target area; (**b**) image from scanning results.

First, to verify the function for 3D point cloud generation using the 2D LiDAR prototype, a test scan was conducted on a small area around a laboratory door. Figure 10 displays a photograph of the scanned area and a 3D model image generated using the scanned point cloud data, for which the 2D LiDAR rotation frequency was set to 40 Hz and the mover speed to 0.5 m/s. As shown in the figure, the LiDAR has a resolution such that not only the shape and depth of the walls and door but also the door handles are clearly recognizable.

**Figure 11:**
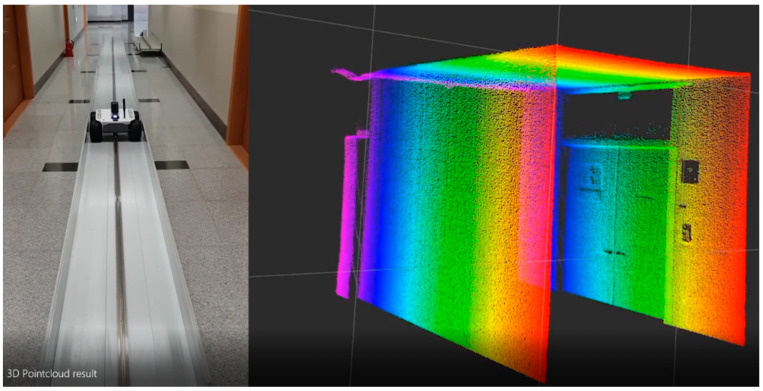
Image of the preliminary test of LiDAR scanning and the result.

Next, the rail described in Section 3 was installed in a building corridor approximately 2.2 m wide and 3 m high in an environment similar to a tunnel tube. A test was carried out in the corridor to generate 3D point cloud models for a space with a length of 15 m. Figure 11 displays images of the test and the results.

**Figure 12:**
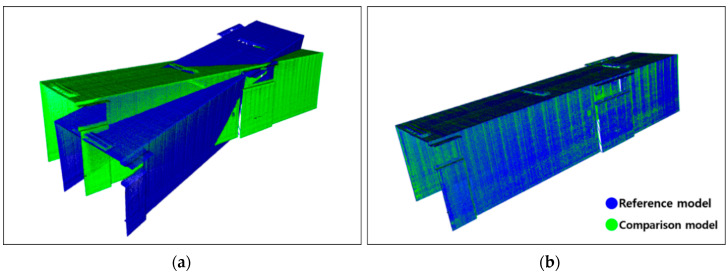
Result of 3D model registration: (**a**) two-point cloud data before registration; (**b**) registered point cloud data.

This test was repeated to generate several 3D models; thus, the model’s registration function was verified. Figure 12 illustrates an example of the result of aligning the orientations of the two models using the registration algorithm developed in this study.

**Figure 13:**
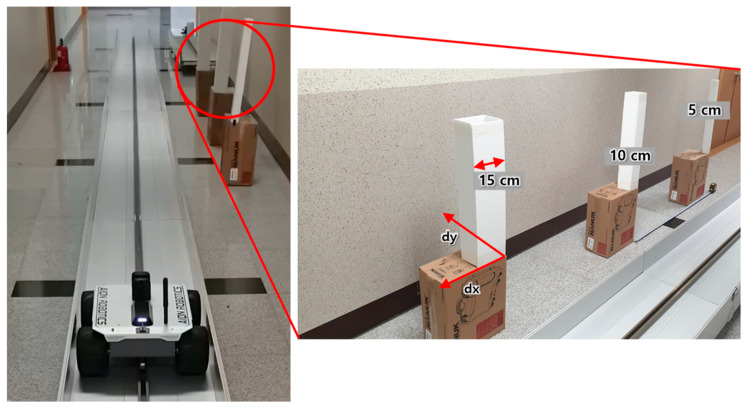
Experiment setup for anomaly detection test of the pilot safety monitoring system.

Considering the above preliminary test results and the performance of the LiDAR sensor (rotation frequency, angular resolution, distance measurement precision, etc.), the experimental environment to test the facility change and anomaly detection function was configured as follows (Figure 13):

- Experiment purpose: change the position of objects and generate 3D point cloud models to detect the changes;
- Objects to change positions: square pillars with widths of 5, 10, and 15 cm;
- Directions and distances of object position change
  ⮚ *x*-axis direction: 0, 2.5, 5 cm
  ⮚ *y*-axis direction: 0, 5, 10 cm;
- Change in rotation frequency of LiDAR sensor: 20, 40 Hz;
- Change in robotic mover speed: 0.3, 0.5, 1.0, 1.7 m/s.

**Figure 14:**
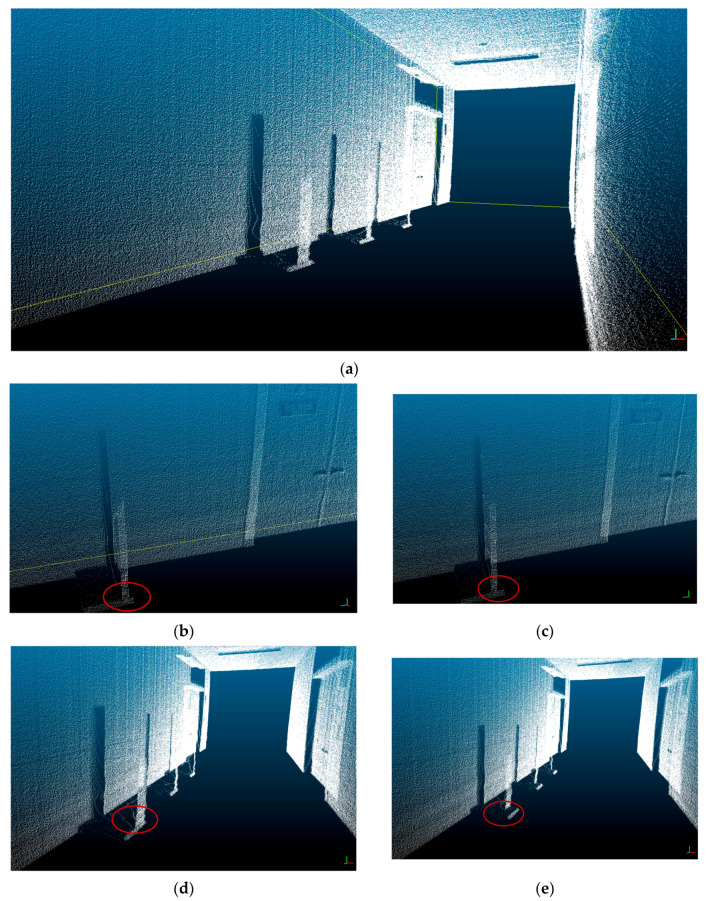
Three-dimensional point cloud modeling result: (**a**) three-dimensional modeling result (reference model); (**b**) moved by 2.5 cm (*x* axis) 2.5; (**c**) moved by 5.0 cm (*x* axis); (**d**) moved by 5 cm (*y* axis); (**e**) moved by 10 cm (*y* axis).

**Figure 15:**
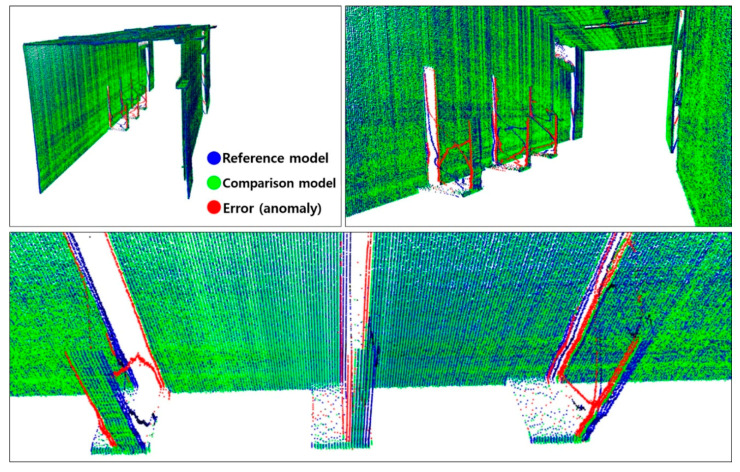
Three-dimensional point cloud model comparison and anomaly detection results.

Figure 14 and Figure 15 demonstrate the experimental results. Figure 14 shows the result of the 3D model generation when the LiDAR rotation frequency is 40 Hz and mover speed is 0.3 m/s. Figure 14 (a) is the reference model (no change), (b) and (c) are comparison models generated after moving the objects 2.5 and 5 cm forward in the *x*-axis direction, respectively, and (d) and (e) are comparison models generated after moving the objects 5 and 10 cm forward, respectively, in the *y*-axis direction.

Figure 15 compares the 3D point cloud models and anomaly detection results using the visualization module developed in this study. The comparison reveals that the change detection algorithm sensed the changes in shape with respect to the reference model and the comparison model generated with the sensor setting of 40 Hz rotation frequency and 0.3 m/s mover speed, after moving the square pillars in 2.5 cm increments in the *x*-axis direction. The detected anomaly is indicated in red, and the change detection threshold value is 20 mm. In the figure, blue indicates the reference model, green indicates the comparison model, and red indicates changes with a difference of 20 mm or more between the two models. As shown, cases in which the position of the square pillars moved by 2.5 cm were properly detected. Tests with different experimental conditions also yielded identical results, confirming that the 3D safety monitoring prototype created in this pilot study can detect anomalies (out-of-position or foreign bodies) of 2.5 cm or more in size. Moreover, it could be assumed that anomalies of approximately 2 cm in size could also be detected, even though tests with this condition were not conducted.

This study used a 2D LiDAR sensor, given that it is a pilot study to confirm the possibility of configuring an anomaly detection system using LiDAR. Enhancing the performance of the LiDAR sensor will not only enable the detection of smaller anomalies but also increase the detection speed, which will be key to enabling the practical use of this technology.

**Figure 16:**
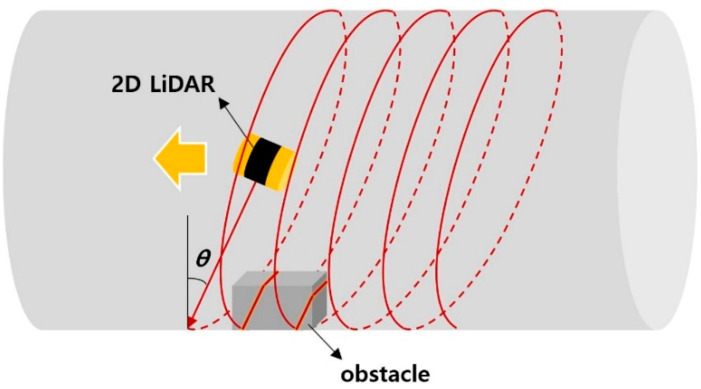
Angle scanning concept with a 2D LiDAR.

Because the LiDAR senses in the direction perpendicular to the *x*-axis which is the movement direction and parallel to the yz-plane, the sensing performance degrades on surfaces parallel to the yz-plane. Moreover, the back side of all surfaces reached by the laser beam could not be sensed due to the shadow effect. To solve these problems, as shown in Figure 16, a setup can be devised in which the yz-plane, i.e., the sensing direction, is tilted forward or backward at an angle rather than being perpendicular to the *x*-axis. This would enable the laser beam to reach not only surfaces perpendicular to the movement direction of the scanning system, but also the spaces behind the objects. Furthermore, by simultaneously using a sensor with a forward-tilted angle and a sensor with a backward-tilted angle, the issue of shadows in the sensor movement direction caused by sensor tilting can be solved. Experiments to verify these techniques should be performed in the future.

The method of combining a LiDAR system and photogrammetry, which is able to extract 3D data information from sets of 2D imagery, could improve scanning performance. This combination could avoid problems such as occlusions and data gaps, which frequently arise when using LiDAR alone. A future study is being prepared to apply this method.

Research is also necessary to verify the usability of laser scanning systems in a vacuum state, such as hyperloop tubes, rather than general facilities in the air. This is because it is impossible to air-cool the heat generated in a system in an environment with a scant amount of air, and heat generation must be minimized in a sealed tube. It is also necessary to confirm whether the control board’s circuit will properly operate in a vacuum state and whether a solution for this will be required.

The safety monitoring system developed in this pilot study will be suitable for automatic, quick, and frequent monitoring for the presence of potential hazards to vehicle safety in a relatively wide area, such as the space in which vehicles drive inside a tube. Accordingly, methods such as laser profiling or structured light scanning may be more appropriate for the precise inspection of local areas, such as rail integrity or damage inspection at expansion joints.

A hyperloop uses a closed tube in a near-vacuum state in which pod-type vehicles move at 1000 km/h or more, which distinguishes this technology from maglevs and all other existing transportation modes. This study provides an automated 3D safety monitoring system for facilities with such characteristics, which is a significant step in the development and realization of hyperloops.

## 5. Conclusions

In a hyperloop, vehicles travel at high speeds inside a sealed tunnel tube facility in a near-vacuum state. This concept has characteristics distinct from existing transportation systems. Various equipment such as communication- and electricity-related facilities for driving the vehicle should be installed around the circular wall inside the tube. In this regard, it is imperative that the driving path of vehicles moving at high speeds inside the facility is clear of obstacles. Therefore, it must be possible to rapidly and automatically monitor the entire 3D space inside the tube to swiftly detect potential hazards in the driving path in real-time. Accordingly, as a solution, a pilot study was performed to verify the applicability of an automated anomaly-detection technique based on 3D point cloud models using a LiDAR sensor.

For this study, a prototype 2D LiDAR sensor with a rotation frequency of 40 Hz, a field of view of 180°, an angular resolution of 0.1°, and pulse repeatability of 75 kHz, was fabricated. For rail scanning tests, a robotic mover and a travel rail 15 m long were manufactured. A dedicated viewer for transmitting, storing, and visualizing the analytical results of data was developed and designed to execute all the tasks.

The tests on the prototype developed in this study demonstrated that anomalies such as a 25 mm change in position or the introduction of foreign objects were accurately detected. The 2D LiDAR-based 3D safety monitoring system developed in this study can detect anomalies in the internal conditions of tube facilities in real-time and help efficiently initiate the necessary measures, thereby enhancing safety. It can also be utilized as a foundation for automating the collection of facility condition data, paving the way for the use of artificial intelligence to implement decision-making, for the efficient evaluation of facility health in the future. For these reasons, the system developed in this study is highly significant.

By improving the sensing method in the future, such as by utilizing sensors with better performances than the 2D LiDAR in this study, or by applying intersection angle sensing, it should be possible to enhance the speed and precision of anomaly detection, thereby increasing the system usability.
